# Tourism under the Early Phase of COVID-19 in Four APEC Economies: An Estimation with Special Focus on SARS Experiences

**DOI:** 10.3390/ijerph17207543

**Published:** 2020-10-16

**Authors:** Bao-Linh Tran, Chi-Chung Chen, Wei-Chun Tseng, Shu-Yi Liao

**Affiliations:** Department of Applied Economics, National Chung Hsing University, Taichung 402, Taiwan; linhtran.ymn@gmail.com (B.-L.T.); tweichun@nchu.edu.tw (W.-C.T.); sliao@nchu.edu.tw (S.-Y.L.)

**Keywords:** coronavirus, COVID-19, SARS, international tourism, panel data model, fixed effects

## Abstract

This study examines how experience of severe acute respiratory syndrome (SARS) influences the impact of coronavirus disease (COVID-19) on international tourism demand for four Asia-Pacific Economic Cooperation (APEC) economies, Taiwan, Hong Kong, Thailand, and New Zealand, over the 1 January–30 April 2020 period. To proceed, panel regression models are first applied with a time-lag effect to estimate the general effects of COVID-19 on daily tourist arrivals. In turn, the data set is decomposed into two nation groups and fixed effects models are employed for addressing the comparison of the pandemic-tourism relationship between economies with and without experiences of the SARS epidemic. Specifically, Taiwan and Hong Kong are grouped as economies with SARS experiences, while Thailand and New Zealand are grouped as countries without experiences of SARS. The estimation result indicates that the number of confirmed COVID-19 cases has a significant negative impact on tourism demand, in which a 1% COVID-19 case increase causes a 0.075% decline in tourist arrivals, which is a decline of approximately 110 arrivals for every additional person infected by the coronavirus. The negative impact of COVID-19 on tourist arrivals for Thailand and New Zealand is found much stronger than for Taiwan and Hong Kong. In particular, the number of tourist arrivals to Taiwan and Hong Kong decreased by 0.034% in response to a 1% increase in COVID-19 confirmed cases, while in Thailand and New Zealand, a 1% national confirmed cases increase caused a 0.103% reduction in tourism demand. Moreover, the effect of the number of domestic cases on international tourism is found lower than the effect caused by global COVID-19 mortality for the economies with SARS experiences. In contrast, tourist arrivals are majorly affected by the number of confirmed COVID-19 cases in Thailand and New Zealand. Finally, travel restriction in all cases is found to be the most influencing factor for the number of tourist arrivals. Besides contributing to the existing literature focusing on the knowledge regarding the nexus between tourism and COVID-19, the paper’s findings also highlight the importance of risk perception and the need of transmission prevention and control of the epidemic for the tourism sector.

## 1. Introduction

As the world has become increasingly interdependent and connected, disasters and crises that happen in one single place can significantly cause general economic and tourism specific effects to a broader area or worldwide. During the past decades, the tourism industry has suffered multiple disruptive events, including the outbreak of foot and mouth in the United Kingdom (2001), the September 11 terrorist attack (2001), the Indian Ocean earthquake and tsunami (2004), and the global economic crisis in 2008/2009, etc. Also, epidemics, such as the epidemic of the severe acute respiratory syndrome (SARS) of 2003 or the Middle East respiratory syndrome (MERS) outbreak in 2015, which are considered as public health crises, have huge adverse impacts on international tourism.

The vulnerability of tourism to political and economic disruptions and disasters has long been evident. The magnitude of effects or resilience capacity is different depending on the spatial and temporal scale of crisis. The 26 December 2004 Indian Ocean earthquake and tsunami, which is considered one of the worst natural disasters, occurred in Sumatra, Indonesia, caused serious damage to many countries in and around the eastern Indian Ocean. In Thailand, there were 50 different nationalities among the deceased, and many lost were international visitors [1]. After the disaster, there has been a significant decline in international arrivals to the country, which is also reflected by a huge reduction, by 50–70%, in the hotel occupancy rate in 2005, compared to the same period of 2004 [2]. The September 11 terrorist attack (2001) in New York was found to cause a shock on tourists’ utility. After the terrorist attack event, all attributes made a lower contribution to visitor’s utility and to the attractiveness of related destinations [3]. At a larger scale, the tourism sector experienced numerous challenges as a result of the global economic crisis in 2008/2009. While the crisis was found to cause negative impacts on inbound and outbound tourism in Asia and would rebound from 2010 onwards [4], it has also been estimated to have led to a 4% decline in international arrivals globally [5].

Regarding the damages to international tourism caused by public health crises, the epidemic of SARS of 2003 is calculated to cause a reduction of 8.6 million international arrivals or 1.2% from the year of 2002 [6]. The impact is found more serious especially in Asia and the Pacific in that there was a drop of 12 million arrivals, constituting a 9% drop of the year before [7]. More specifically, the negative effects of the epidemic on country-specific international tourism demand are found ranging from 175 to 1742 as one more person infected by SARS [8]. The 2004 Avian influenza (H5N1) outbreaks caused total economic losses in Southeast Asia around US$10 billion [9], while Asian tourism demand is estimated to be reduced by 168 cases for each additional outbreak in poultry [10]. Another epidemic is Ebola, which peaked in the period of 2013–2014. Besides causing a wider uncertainty, the disease outbreak has been also recognized as creating negative perceptions for African destinations that were unaffected by Ebola [11,12]. More recently, the emergence of the MERS outbreak in 2015 significantly afflicted South Korea tourism. In particular, even though MERS does not influence domestic tourism demand, the disease is found to be correlated with a reduction of 2.1 million non-citizen arrivals corresponding with US$ 2.6 billion in tourism loss [13]. Most of these crises caused a short-term decline in the global and local development of tourism. This would suggest that tourism as a system has been resilient to external shocks.

More recently, an infectious disease caused by severe acute respiratory syndrome coronavirus 2 (SARS-CoV-2) [14], otherwise known as COVID-19 [15], spread from Wuhan, Hubei province of China (December 2019), has rapidly resulted in a pandemic all over the world. The first case of COVID-19 outside China was reported on January 13. As of April 30, 2020, 3,090,445 cases have been confirmed in 215 countries and territories [16]. Some 191 of these countries have experienced local transmission and there have been mortalities in at least 166 of them. Despite the lower case mortality rate compared with SARS and MERS, the overall number of confirmed cases and deaths from COVID-19 far outweigh those from the other two epidemics [17]. A person infected with COVID-19 could go unrecognized, experiencing very mild, delayed, or no symptoms. In that way, the undocumented but infectious case is one of the major epidemiological characteristics that caused the pandemic potential of the emergent coronavirus, spreading from China to all around the world within just 3–4 months [18].

In efforts to contain the transmission of the virus, especially when vaccines and therapeutics are not yet available, physical distancing measures and movement measures have been increasingly applied. With many countries imposing international and local travel ban, closing borders, or introducing quarantine periods, the tourism sector has been hit heavily. According to an estimation by the UN World Tourism Organization (WTO), the COVID-19 crisis was projected to cause a 60–80% decline in 2020 international arrivals relative to 2019 globally [19]. The disease may put 100 million jobs in the global travel and tourism sector at risk, and countries that are heavily exposed to the pandemic were the hardest hit [20,21].

The nexus between COVID-19 and tourism have been approached under different perspectives. The majority of research has estimated the effects of the COVID-19 pandemic on travel and tourism, where impacts on the aviation industry, tourism, and socio-economy were assessed [21,22,23,24,25,26] and the willingness to pay to reduce negative effects of the pandemic were estimated [27,28]. Some studies suggested potentials of interdisciplinary research collaboration with regards to COVID-19 [29] and discussed the relationships between tourism and sustainable development through the lens of the crisis [30]. Tourism, by the nature of its system, has experienced the impacts of COVID-19 but it has originally contributed to the spread of the disease. Thus, some papers, in contrast, have accessed the effects of the travel and tourism on the number of COVID-19 cases [31,32] or estimated the effectiveness of travel restrictions on the spread of coronavirus disease [33,34].

The impacts of COVID-19 on the tourism industry could be observed or projected. The majority of researchers have approached the tourism effects of COVID-19 with observed influences. Using daily data of global COVID-19 cases and flight volumes, Gössling et al. [21] have visually represented the negative impact of COVID-19 on the number of global flights. The study also compared the impacts of COVID-19 to previous global infectious diseases and explored the effects of the pandemic on the socio-economy and tourism. At a national level, the impact of the pandemic on aviation industry and tourism accommodation in Malaysia are accessed by Karim, Haque [23]. Dinarto, Wanto [25] reported the impacts of the crisis on tourism sector in Bintan, an island in the Riau Archipelago, Indonesia, while another study conducted by Correa-Martínez, Kampmeier [24] discussed the spread of the coronavirus in a skiing area in Austria. The observed impacts of COVID-19 on tourism are basically conducted by using descriptive statistic methods.

In terms of the impacts projected, a 20 May 2020 press release from the UN World Tourism Organization (WTO) estimated the pandemic would cause a 60–80% loss in international tourist arrivals [19] rather than the forecasted 20–30% decline that was published two months earlier on 26 March [35]. The uncertainty in the predicted impact reflected the continuously unprecedented evolutions of the COVID-19 pandemic during the first half of 2020. In a semi-annual report, the International Air Transport Association (IATA) estimated that the revenue passenger kilometers (RPK) will be decreased by 54.7% compared with 2019 [36]. Iacus, Natale [22] predicted that with the COVID-19 pandemic, in the worst scenarios, travel restriction can impact 1.67% of the overall GDP, generating a loss of US$ 323 billion and 30.3 million job losses worldwide. The World Travel & Tourism Council (WTTC) [20] has estimated the crisis to cause much higher losses, namely over 100 million tourism job losses and total economic losses of up to US$ 2.7 trillion in the year of 2020. The projected impacts are calculated by analyzing data and metadata provided in relation to regular estimates published by tourism organizations.

Projection is a statistic indicating what a value would be if the assumptions about the future hold true. If these estimates are not interpreted with extreme caution, especially for an uncertain development of the crisis, coping strategies could be orientated ineffectively. Unlike methodologies used in projecting the tourism impacts of COVID-19, regression approach to make predictions does not necessarily involve predicting the future. Instead, we predict the mean of the dependent variable given specific values of the independent variables. This approach allows us to quantify the effects from observed variables and some disturbances, test whether the parameters are statistically significant, then use them for prediction and policy purposes. In this study, the relationship between COVID-19 and tourism will be better clarified with econometric models applied.

Being considered a direct predecessor of COVID-19, SARS was first identified in Guangdong, China in November 2002, which then later spread over many Asia countries and regions. Outside of China, Hong Kong and Taiwan experienced the most serious impacts from the SARS outbreak, with 1755 cases and 299 deaths in Hong Kong and 346 cases and 73 deaths in Taiwan [37]. The two economies have put lessons they learned from the 2003 SARS outbreak to good use, with a quicker and more effective response when COVID-19 emerged. As of April 30, when there were more than 3 million infections reported over the world (an average of about 14,000 cases per country), Hong Kong and Taiwan only had 1036 and 430 confirmed cases, respectively. Even though the effect of travel restrictions is immediate and undeniable, not all economies put in place a travel ban quickly and simultaneously. During the time gaps, the magnitude of the impacts of COVID-19 on the number of international arrivals may be significantly different among tourist destinations, which depends on how a destination is perceived as safer.

In order to contribute to a better understanding of the effects of COVID-19 on tourism, with a special investigation on SARS experiences, our study applies panel data regression models to estimate the epidemic-tourism relationship for four countries/economies, including Taiwan, Hong Kong, Thailand, and New Zealand. Specifically, we (1) quantify impacts of the number of COVID-19 confirmed cases on the number international tourist arrivals and (2) decompose data set into different groups of economies to access the differences in impacts of COVID-19 pandemic on tourism between groups with and without SARS experiences.

In the following sections, we provide the theoretical and empirical model structure for constructing the relationship between related-COVID-19 variables and tourism. Section 3 presents the estimation results and discussion. Finally, policy implications and concluding remarks are provided in Section 4.

## 2. Methodology

### 2.1. Panel Regression Model

In this article, panel regression model is employed to depict the relationship between COVID-19 pandemic and international tourist arrivals. A multiple panel regression model is assumed as follows:

(1) $$Y_{it}\;=\;\alpha \;+\;\beta^{\prime }X_{it}\;+\;u_{it},$$

where i and t denote, respectively, indexes of the individual (economy) and the time (date), Y is tourist arrivals, and X is a set of independent variables, e.g., the number of confirmed cases, the number of global deaths, exchange rate, and a dummy variable for travel restriction policy. The estimation of the parameters gives the average effect of the explanatory variables on tourism demand. $u_{it}$ is the error term in two components $u_{it}$ = $\mu_{it}$ + $v_{it}$, where $\mu_{it}$ is the unobservable individual-specific effect and $v_{it}$ is the random disturbance (for further details, see Baltagi 1995 [38]).

Before conducting the tourism demand function, it is important to establish the correct panel form. The Hausman test is applied to test for fixed and random effects under the null hypothesis ($H_{0}$), which states that $\alpha_{i}$ is not correlated with $X_{it}$, in other words, the random effects estimator is consistent and efficient. On the other hand, the alternative hypothesis ($H_{1}$) supposes that the random effect estimator is inconsistent.

The empirical panel data model is given in natural logarithm form as follows:

(2) $$\ln\;Arrivals_{it}\;=\;\alpha_{0}\;+\;\beta_{1}\ln\;COVID\_case_{it-7}\;+\;\beta_{2}\;\ln\;ER_{it}\;+\;\beta_{3}\;\ln\;Travel\_ban_{it}\;+\;u_{it},$$

The number of COVID-19 confirmed cases (*COVID_case*) and the date at which the travel restriction policy (*Travel_ban*) came into effect in the tourist destination are considered to be major pandemic-related variables. As a travel restriction is considered one of the most effective policies for mitigating the virus transmission starting from imported cases, in comparison with other measures [33], it was used in our estimation. *Travel_ban* is dummy variable that equals 1 from the date the travel restrictions take effect. As far as the influencing factors are concerned, we also consider the foreign exchange rate (*ER*) an important determinant of tourism. *ER* is the amount of money in the tourist destination currency that is required to exchange to one US dollar. Thus, the variable is expected to cause a positive effect on the demand of tourism. Finally, as people usually plan to travel several days or weeks before departures, time lag effect is also included. In this article, we use a lag period of 7 days for the information on daily COVID-19 confirmed cases in destination countries or regions. All analyses are conducted using Stata version 14.0.

### 2.2. Data Set

We obtain daily data for daily COVID-19 confirmed cases and the number of international tourist arrivals from different official websites of each economy. Due to the availability of data, the sample economies are Taiwan, Hong Kong, Thailand, and New Zealand and the time period we use is 1 January–30 April 2020, except for data of tourist arrivals to Hong Kong (24 January–30 April 2020). Tourism data of Hong Kong is the total daily number of arrivals of non-Hong Kong residents. Data on tourist arrivals to Taiwan and Thailand are passenger volume in Taoyuan airport (total number of passengers) and Bangkok airport (foreign nationals), respectively. For New Zealand, a large file containing all information for very single movements across New Zealand borders is retrieved and filtered for daily data of the total arrivals of non-residents to New Zealand. Data sources are specified in Table 1.

Data on the exchange rate are retrieved from the webpage of Economic Research Division of Federal Reserve Bank of St. Louis. In response to the continued spread of COVID-19, Taiwan and New Zealand barred foreign nationals from entering their territory starting March 19; Hong Kong closed borders to all non-residents from 25 March; and Thailand put the travel restriction into effect starting 29 March. The summary statistics are presented in Table 2, which shows that higher means are correlated with higher standard deviations. The variations in tourist arrivals in Hong Kong are more dramatic than the variations in the three remaining. Among the four selected tourist destinations, the average number of COVID-19 confirmed cases in Thailand was highest at about 24 cases per day, followed by New Zealand (9 cases per day), Hong Kong (8 cases per day), and Taiwan (4 cases per day).

## 3. Estimation Results

### 3.1. The Impacts of Coronavirus Disease (COVID-19) Pandemic on International Tourism

Three estimation approaches for Equation (2), including Pooled OLS, fixed effects (FE) model, and random effects (RE) model, are run to deal with common panel data problems such as heteroscedasticity, autocorrelation, or cross-correlation in cross-sectional units at the same point in time. The results of F test and Lagrange Multiplier (LM) test indicated that FE model and RE model are both better than the pooled OLS model. The Hausman test was applied to test for fixed or random effects. Based on the Hausman test result, we can conclude that fixed effects model is the most appropriate model for our panel data.

Table 3 displays the results of all panel regression models with three explanatory variables only since the RE estimation procedure requires the number of cross-sections to be greater than the number of coefficients. As the Hausman test result shows that FE model is more appropriate, we add one more independent variable, i.e., the daily number of global confirmed COVID-19 deaths, into the FE model. The parameter estimates are presented in Table 4.

Since the logarithmic form was adopted, the coefficients displayed in Table 3 and Table 4 are presented as elasticities. Table 4 shows that the coefficients of national confirmed cases and global deaths are all negative and significant. This indicates that the tourism industry in the four sample economies has been decimated by COVID-19 over the first four months of the year 2020. At the 1% level of significance, we find that a 1% COVID-19 confirmed case increase causes a 0.075% decline in tourist arrivals. By multiplying the estimated coefficient times by average dependent variable divided by the average number of confirmed cases, the estimate implies that the daily tourist demand was reduced by about 110 arrivals for an additional person infected by COVID-19. A 1% increase in the number of global deaths caused by COVID-19 resulted in a lower passenger volume by 0.049%. It is worth noting that the impact of travel restrictions on tourism is found to be very significant and negative. Specifically, the average number of international tourist arrivals after the travel ban came into effect in the four tourist destinations is lower than prior periods, by about 321.5%. Finally, sign of exchange rate coefficient is found to be significantly negative, which implies that as the value of destinations’ currency decreases, the tourism demand still keeps reducing. This indicates that the tourism markets are exhibiting a lower sensitivity to price changes among tourists selecting the four sample economies as their destinations.

### 3.2. The Effects of COVID-19 on Tourism in Economies with and without Experiences of SARS-CoV Epidemic

Lessons learned from 2003 SARS experiences have helped Taiwan and Hong Kong’s governments to establish an effective public health response mechanism for quickly enabling actions to deal with COVID-19, leading to a very low rate of indigenous transmission in these two Northeast Asia economies. During the epidemic time, the image of such tourist destinations is therefore perceived as safer, in comparison with others without or less SARS experiences. From that perspective, the impacts of the COVID-19 outbreak on international tourism is also expected to differ when tourist destinations’ SARS experience is considered. The study goes one further step to estimate the effects of COVID-19 on the number of tourist arrivals in groups of economies with and without experiences and risk perception of pandemic. Data is now decomposed into two different sets: the first set includes Taiwan and Hong Kong, while the second set includes Thailand and New Zealand. In fact, New Zealand and Thailand both had people infected by the SARS outbreak of 2003. However, as these numbers are very small compared with Hong Kong or Taiwan, Thailand and New Zealand are still considered to have less or no SARS experiences. Additionally, according to estimates from the World Travel & Tourism Association [7] in 2019, the contribution of tourism sector in Thailand and New Zealand to their GDP and total employment were higher than those in Hong Kong and Taiwan. From this perspective, the grouping approach is appropriate, relating the consistency in term of the reliance on tourism of each country or region. Fixed effects model is then applied for these two data sets, since (1) the FE model can control for the unobserved heterogeneity and time-invariant issues in comparison with OLS and (2) the model is tested prior to ensure it captures the pandemic-tourism relationship better than RE models. The effects of COVID-19 on tourism demand in two groups of economies estimated using FE models are presented in Table 5.

Related-COVID-19 epidemic coefficients are all found to be significant and negative. However, the magnitude in relation to national cases and global deaths on international tourism within each group and between two groups differed significantly. First, the impact of COVID-19 on tourism in the destinations without SARS experience is found much more severely in comparison with the tourist destinations that have experienced the pandemic. The estimation results indicate that the average tourist arrivals to Taiwan and Hong Kong decreased by 0.034% in response to a 1% increase in COVID-19 confirmed cases, while in Thailand and New Zealand, a 1% national confirmed cases increase caused a 0.103% reduction in tourism demand.

Second, we find that the tourism effect of global COVID-19-caused mortality on international tourism in Taiwan and Hong Kong is stronger than the effects of the number of confirmed cases. For Thailand and New Zealand, the average number of arrivals to a country is influenced by the information about the number of COVID-19 cases in that country, much more than by global pandemic-related mortality. This finding is indicative of a transitive effect of experiences of the SARS epidemic of 2002. As Taiwan and Hong Kong suffered serious losses from SARS, the governments have a quicker and more effective response to the COVID-19 outbreak than other economies with no or less experience with SARS, such as Thailand and New Zealand. This results in how Taiwan and Hong Kong have kept their coronavirus infection rate very low despite their proximity to China. A low infection rate would certainly be one of the major reasons that tourism in these economies tended to be affected more by information about global COVID-19 deaths than by the number of domestic confirmed cases.

Third, exchange rate coefficients are only found to be significantly positive for the first group, which means that currency depreciation in Taiwan and Hong Kong would encourage increasing tourism demand, and vice versa. Finally, travel restriction policy in response to the spread of the COVID-19 pandemic is found to be the major influencing factor for tourism in both groups. However, the effect of a travel ban on tourism decline in Thailand and New Zealand was much stronger than in Taiwan and Hong Kong.

## 4. Conclusions

As the tourism sector is one of the hardest-hit by the outbreak of COVID-19, a clear understanding of the relationship between the crisis and tourism demand is critical for the identification of appropriate adaptation strategies to minimize the negative impact of COVID-19 on the economy in general, as well as the tourism industry in particular. In light of the importance of identifying the effects of the COVID-19 pandemic on international tourism, this study contributes to the existing literature the quantified tourism impacts of COVID-19, under the view of SARS experiences, using econometric modeling approach. 

The panel regression model results demonstrate how COVID-19 could significantly devastate international tourism for four APEC economies. The estimated average negative effect of the COVID-19 epidemic on international tourism is a decline of approximately 110 arrivals for an additional person infected by the coronavirus, during the first four months of the year 2020. As the demand for international tourism is significantly affected by the destination countries’ health security, the magnitude of negative impacts of COVID-19 on international tourism would be different between two groups, namely those with and without SARS experiences. Our estimation result reveals that the impacts of domestic COVID-19 cases on international tourist arrivals to Taiwan and Hong Kong were less severe than those in Thailand and New Zealand. For instance, the average tourist arrivals to Taiwan and Hong Kong decreased by 0.034% in response to a 1% increase in COVID-19 confirmed cases, while in Thailand and New Zealand, a 1% national confirmed cases increase caused a 0.103% reduction in tourism demand. Under the same combination of variables in estimating two groups, international tourism demand was influenced by the number of global COVID-19 deaths more than by domestic confirmed cases for SARS-affected economies, but was affected more severely by local COVID-19 cases rather than global mortality in case of non-SARS-affected economies.

The findings of disproportionate impacts of related COVID-19 factors on tourism demand for the two nation groups suggest that hard-won lessons from the past could help governments in retaining risk perception of pandemic to combat the new coronavirus quickly and effectively. As COVID-19 has debilitated the tourism industry worldwide, keeping the destination at a very low infection rate may not protect the tourism sector during the epidemic time. However, this advantage not only assists the economies to minimize the general economic damage but also can definitely help domestic tourism sector to restore more quickly when the pandemic is under control.

In response to the global spread of COVID-19, travel restriction is one of the most effective isolated intervention implemented to slow down the dispersion elsewhere in the world, especially when governments respond rapidly. In this study, we find that travel restriction on tourist arrivals has a larger effect in Thailand and New Zealand, compared to Taiwan and Hong Kong. This result could be explained based on the delay in responding to the pandemic from Thai government. In Thailand, tourism has become one of the only sources of foreign exchange earnings since 1997 [47] and has experienced phenomenal growth in tourist arrivals in recent years. During the study period, Thailand had the largest number of domestic COVID-19 confirmed cases compared to the other three economies. However, while Taiwan, New Zealand, and Hong Kong have prohibited foreign visitors from entering their boundary from 19 March and 25 March 2020, Thai government still opened borders until 29 March. Thus, the underlying reasons for the government’s procrastination in imposing a travel restriction could be the lack of pandemic experiences and the heavy dependence of the economy on international tourism. Based on the estimation results, all governments, even tourism-dependent economies, are suggested to take swift actions to contain the spread of the virus, since a delay in response could lead to direct impacts, such as uncontrollable virus transmission, which can cause negative effects for the tourism sector and the whole economy.

### 4.1. Limitations of the Study and Recommendations for Future Research

The study has several limitations that could be further explored in future research. First, although New Zealand is grouped as a country that has not had much experiences with SARS, and is explained in the same trend of effects as Thailand, the country has thus far appeared to have fared better than Thailand in controlling the disease, with a small number of cases and low transmission rate. In other words, beside the difference in experience of SARS, the effects of COVID-19 on tourist arrivals in the two nation groups also represent the difference in many other influencing factors, such as health care systems, governments’ efficiency in taking response actions, tourism market size, and structure of tourists’ source countries, etc. Thus, further research is encouraged to consider the nexus between pandemic and tourism more comprehensively to achieve better estimation results. Second, the international tourist arrivals are not only affected by travel restrictions of destination country, but also affected by the time the source countries or regions started limiting their own residents from traveling abroad. Therefore, other approaches that consider travel restriction policy more thoroughly could be useful in providing more appropriate estimates. Third, due to data availability, the number of cross-sections in panel data model was limited to four. Sample countries of non-SARS-affected group could be better selected (countries with large tourism market size, completely no experience of SARS, and severely affected by COVID-19) to investigate the pandemic-tourism relationship through the lens of SARS experience more in-depth. Approaching a larger and better dataset is encouraged in future research. Finally, though tourism industry is usually considered to be resilient, bouncing back relatively quickly after significant events, the effects of COVID-19 on tourism could differ significantly from other crises, as there many potential risks of surging other outbreaks remain once travel restrictions are eased or removed. At the time of writing, a rise in locally transmitted cases in Hong Kong has been recorded, the number of COVID-19 infections worldwide have exceeded 30 million and deaths have surpassed 1 million, and unemployment figures have risen steeply in many countries. Hence, the impact and recovery from the COVID-19 pandemic could be unprecedented and require to be studied more intensively in the future.

### 4.2. Taiwan’s Response to COVID-19

On December 31, 2019, when China reported 27 cases of the unidentified viral pneumonia outbreak to the WHO [48], the Taiwan CDC initiated health checks onboard flights from Wuhan. On January 5, 2020, all travelers from Wuhan in the past 14 days that had a fever or symptoms of upper respiratory tract infection would be screened for 26 viruses, including SARS and MERS [49]. The health screening measures have been gradually expanded to flights from China, and then to all incoming flights (by March). In response to a growing number of infections caused by a COVID-19 in neighboring countries, on January 20, 2020, the Taiwan CDC announced the activation of the Central Epidemic Command Center (CECC) [50], which has played an important role in recognizing and controlling the health crisis of COVID-19. During the first 50 days of the COVID-19 outbreak, the CECC coordinated comprehensive efforts by various ministries to manage the health crisis to implement more than 100 actions regarding border control, case identification, home isolation or quarantine, proactive case finding, manufacture and allocation of masks, and strategies for enhancing quality of education and reassurance of the public [49,51].

The experiences from SARS, beside generating instrumental lessons in disease control measures and policy planning for Taiwan’s government, also effectively improve the public epidemic awareness toward the COVID-19. For instance, compared to other economies, the COVID-19 online information-seeking behavior appeared very early in Taiwan [52], which is very important for public health, as people realize the severity of COVID-19 and proactively increase their epidemic awareness. More importantly, citizens and residents of Taiwan strictly adhere to the government’s health guidelines to prevent the spread of coronavirus. The unified support of the public is revealed by significant changes in health behaviors and hygiene practices, such as frequent hand-washing, use of sanitizers, and wearing masks outdoors, especially when using public transportation.

Thus, as a result of the robust system of government agencies and hospitals and public acceptance of protective measures, policies response to COVID-19 is implemented rapidly in the initial stages of the outbreak COVID-19, helping Taiwan to contain the epidemic effectively. As Taiwan has gone many consecutive days with zero new cases of local infection, while maintaining tight controls at the borders, the Taiwan government has planned to relax COVID-19 control across their boundary and launch economic stimulus packages to revive retailers, spur consumption, and promote domestic tourism.

## Figures and Tables

**Table 1:** Summary of data sources.

Variables (Definition)	Data Sources
COVID-19 cases (daily number of COVID-19 confirmed cases)	Taiwan CDC (Centers of Disease Control) [39] Hong Kong Government Open Data Platform [40] Thailand DDC (Department of Disease Control) [41] New Zealand Ministry of Health [42]
Tourist arrivals (daily number of international tourist arrivals)	Taiwan Taoyuan International Airport Co. Ltd. [43] Hong Kong Immigration Department [44] Thailand Immigration Bureau [45] New Zealand’s official data agency [46]

**Table 2:** Descriptive statistics of variables.

Variables	Mean	Std. Dev.	Min.	Max.
Taiwan				
Tourist arrivals (person)	23,305.79	23,438.25	69	68,663
COVID-19 cases (case)	3.554	6.134	0	27
Exchange rate (NTD/USD)	30.097	0.141	29.72	30.45
Travel restriction	0.355	0.481	0	1
Hong Kong				
Tourist arrivals (person)	6233.63	11,875.14	66	59,577
COVID-19 cases (case)	8.578	14.711	0	65
Exchange rate (HKD/USD)	7.766	0.0125	7.749	7.795
Travel restriction	0.306	0.463	0	1
Thailand				
Tourist arrivals (person)	27,390.01	25,744.83	42	75,249
COVID-19 cases (case)	24.413	40.587	0	188
Exchange rate (THB/USD)	31.613	0.908	30.15	33.04
Travel restriction	0.273	0.447	0	1
New Zealand				
Tourist arrivals (person)	8301.298	6433.496	0	16,830
COVID-19 cases (case)	9.331	20.309	0	95
Exchange rate (NZD/USD)	1.598	0.072	1.494	1.764
Travel restriction	0.355	0.481	0	1
Panel data set				
Tourist arrivals (person)	16,810.29	20,980.1	0	75,249
COVID-19 cases (case)	11.469	25.214	0	188
Exchange rate	17.768	13.299	1.494	33.04
Travel restriction	0.322	0.467	0	1
Global deaths (person)	1878.802	2752.062	0	10,520

**Table 3:** Estimation results of panel regression models.

Variables	Pooled OLS	FE Model	RE Model
Constant	7.622 *** (0.165)	41.503 *** (6.211)	7.654 *** (0.144)
LnCOVID_case_lagged	−0.115 *** (0.011)	−0.082 *** (0.011)	−0.142 *** (0.012)
LnER	0.506 *** (0.048)	−13.837 *** (2.610)	0.502 *** (0.043)
Travel_ban	−3.437 *** (0.155)	−3.342 *** (0.156)	−3.373 *** (0.138)
R-squared	0.775	0.802	0.794
Adjusted R-squared	0.774	0.799	0.793
Root MSE	0.122	0.148	0.152
F test		55.14 ***	
LM test			54.33 ***
Hausman test		57.82 ***	

Note: *** denotes the significance level at 1%. Standard errors in parentheses.

**Table 4:** Estimation results of FE model with an additional variable of Global deaths by COVID-19.

Variables	Estimated Coefficients
Constant	34.412 *** (6.638)
LnCOVID_case_lagged	−0.075 *** (0.012)
LnGlobal_COVID_death	−0.049 *** (0.012)
LnER	−10.732 *** (2.829)
Travel_ban	−3.215 *** (0.160)
R-squared	0.806
Adjusted R-squared	0.802
Root MSE	1.137

Note: *** denotes the significance level at 1%. Standard errors in parentheses.

**Table 5:** Results of panel fixed effects models for two economy groups.

Variables	Taiwan-Hong Kong	Thailand-New Zealand
Constant	−185.938 *** (41.180)	7.524 (8.138)
LnCOVID_case_lagged	−0.034 *** (0.011)	−0.103 *** (0.018)
LnGlobal_COVID_death	−0.096 *** (0.016)	−0.056 ** (0.023)
LnER	70.369 *** (14.831)	0.737 (4.694)
Travel_ban	−2.732 *** (0.140)	−3.943 *** (0.322)
R-squared	0.873	0.802
Adjusted R-squared	0.871	0.797
Root MSE	0.736	1.323

Note: ** and ***, respectively, denote significance at the 5% and 1% levels. Standard errors in parentheses.
